# Monitoring Motor Symptoms During Activities of Daily Living in Individuals With Parkinson's Disease

**DOI:** 10.3389/fneur.2018.01036

**Published:** 2018-12-12

**Authors:** Jenna E. Thorp, Peter Gabriel Adamczyk, Heidi-Lynn Ploeg, Kristen A. Pickett

**Affiliations:** ^1^Department of Mechanical Engineering, College of Engineering, University of Wisconsin-Madison, Madison, WI, United States; ^2^Department of Biomedical Engineering, College of Engineering, University of Wisconsin-Madison, Madison, WI, United States; ^3^Occupational Therapy Program, Department of Kinesiology, University of Wisconsin-Madison, Madison, WI, United States

**Keywords:** Parkinson's disease, activities of daily living, sensors, tremor, bradykinesia, hypokinesia, dyskinesia, freezing of gait

## Abstract

This literature review addressed wearable sensor systems to monitor motor symptoms in individuals with Parkinson's disease (PD) during activities of daily living (ADLs). Specifically, progress in monitoring tremor, freezing of gait, dyskinesia, bradykinesia, and hypokinesia was reviewed. Twenty-seven studies were found that met the criteria of measuring symptoms in a home or home-like setting, with some studies examining multiple motor disorders. Accelerometers, gyroscopes, and electromyography sensors were included, with some studies using more than one type of sensor. Five studies measured tremor, five studies examined bradykinesia or hypokinesia, thirteen studies included devices to measure dyskinesia or motor fluctuations, and ten studies measured akinesia or freezing of gait. Current sensor technology can detect the presence and severity of each of these symptoms; however, most systems require sensors on multiple body parts, which is challenging for remote or ecologically valid observation. Different symptoms are detected by different sensor placement, suggesting that the goal of detecting all symptoms with a reduced set of sensors may not be achievable. For the goal of monitoring motor symptoms during ADLs in a home setting, the measurement system should be simple to use, unobtrusive to the wearer and easy for an individual with PD to put on and take off. Machine learning algorithms such as neural networks appear to be the most promising way to detect symptoms using a small number of sensors. More work should be done validating the systems during unscripted and unconstrained ADLs rather than in scripted motions.

## Introduction

Parkinson's disease (PD) is a common neurodegenerative disease that affects over 1 million people in North America (1). The prevalence increases dramatically with age, with approximately 3% of people over the age of 65 and 10% percent of people over the age of 80 affected (1). Information about individuals with PD has been largely limited to signs and symptoms reported and observed in clinical settings, and findings from laboratory-based research studies. While important to furthering our understanding of the disease, these vantages are often unable to address the complex manner in which the disease affects activities of daily living (ADLs) for the individuals affected or their caregiver(s). To better understand the complex interaction between the individual, their symptoms, the progression of the disease, medication and the activities that occupy their day, observations are needed from within the individual's natural environment. These types of observations require wearable sensors that are easy to put on and take off; do not interfere with the tasks being performed; require little to no maintenance and calibration once in the field; and are sensitive to a variety of relevant signs and symptoms.

The presentation of PD is remarkably heterogeneous between individuals (2). The four cardinal motor features of PD are resting tremor, rigidity, akinesia (or bradykinesia and hypokinesia), and postural instability (3). It is important to have a working definition and understanding of the presentation of these terms in place prior to examining possible instrumentation.

### Motor Symptoms of Parkinson's Disease

Resting tremor typically onsets unilaterally in the distal aspect of the extremities and occurs at a frequency between 4 and 6 Hz. As the name suggests, resting tremor disappears with action and during sleep (3). Rigidity refers to increased resistance present throughout the range of passive movement of a limb (3). Akinesia refers to the absence of movement and includes the well-known phenomenon freezing of gait (FOG). Bradykinesia and hypokinesia refer to slow and small movement, respectively. Postural instability is a notable decline or loss of the ability to maintain an upright posture, which leads to impaired balance and falls. One challenge that has long faced clinicians and scientists studying this population is quantification and unbiased tracking of these primary symptoms, especially during the performance of meaningful activity.

### Clinical Assessment of Parkinson's Disease (PD) Symptoms

Disease severity is primarily assessed using subjective clinical rating scales, most commonly the Unified Parkinson's Disease Rating Scale (UPDRS). In 2007, the Movement Disorder Society published a revised version known as the Movement Disorders Society's Unified Parkinson's Disease Rating Scale (MDS-UPDRS). Both versions use a 0–4 scale in each of several subsections to rate different aspects of PD symptoms (3–5). The revised version addressed the lack of consistency among the 0–4 scales in the various subsections and the low emphasis on non-motor aspects of PD. The MDS-UPDRS has four components: Part I—“Non-motor experiences of daily living”; Part II—“Motor experiences of daily living”; Part III—“Motor examination”; and Part IV—“Motor complications”. ADLs are assessed in Part II via a series of self- or caregiver-reported questions regarding task performance during eating, dressing, hobbies, hygiene, and walking and balance. The motor component (section Results) is completed by a trained rater who assesses individual components of motor function. Section Discussion assesses time spent with dyskinesia, functional impact of dyskinesia, time spent in the “off” state, and functional impact of fluctuations (5). Despite the subjective nature of these assessments, the UPDRS and MDS-UPDRS are regarded as the gold standards of assessment for individuals with PD (4).

### Treatment

The most common treatment for PD is levodopa (L-dopa) therapy (1). However, motor fluctuations (systematic increases in symptom severity as the effect of the medication wanes) occur in approximately 50 percent of patients within 5 years of beginning pharmacological treatment and approximately 70 percent of patients after 15 years (1). This is often debilitating to the affected individual to the extent that daily activities and schedules are set around the fluctuations. Motor fluctuations include “on” and “off” states, where the “on” state refers to when symptoms are better controlled, and the “off” state refers to the reappearance or increase of symptoms and decreased mobility. Additionally, the long-term use of L-dopa therapy commonly results in levodopa-induced dyskinesia during the “on” state. Dyskinesias are defined as involuntary abnormal movements (1). Assessment of dyskinesias via self-report can be problematic because patients are frequently unaware of the presentation and extent of their dyskinesias (6). There are also additional scales used to assess dyskinesias which are not specific to PD. The most widely used scales are the Abnormal Involuntary Movements Scale (AIMS) and the modified AIMS (m-AIMS) (6). A patient-friendly mechanism to observe and monitor dyskinesia, motor fluctuations and other symptoms could serve to drastically improve the long-term care of individuals with PD.

### Wearable Instrumentation

Modern wearable sensors offer the opportunity to improve the objectivity and relevance of the assessment and treatment of individuals with PD by quantifying symptom presentation in settings that are uncontrolled, unscripted and unsupervised. Of particular interest is the possibility of measurement and evaluation during the performance of activities of daily living at home or in the community. Such data could promote a deeper understanding of issues such as how symptom severity affects performance of ADLs, when “on” and “off” periods of motor fluctuations occur, how this relates to falls, and to what extent symptoms affect activity level. Ideally, a wearable monitoring system should be (i) sensitive to change, (ii) accurate, (iii) able to relay data to a remote location, (iv) low maintenance, (v) durable, and (vi) equipped with methodologies to extract information that is clinically relevant from the raw data without being overly cumbersome and intrusive (7). The Movement Disorders Society Task Force on Technology also recently suggested wearable technologies should be developed as open platforms, integrated with medical records, and potentially integrated in treatment delivery systems in order to translate these technologies into better care and management for PD patients (8).

Research investigating home-based monitoring of individuals with PD is currently being conducted and recent participants have reported the computer equipment and sensors were easy to operate. The majority complied with wearing the sensors at home throughout their entire study, suggesting future studies of home-based interventions with continuous activity monitoring using wearable sensors are feasible in the PD population (9, 10).

Sensors that are unobtrusive enough for the participant to wear throughout the day allow for the measurement of mobility including total daily activity level and functional mobility measures of turning and postural transitions. This type of long-term monitoring allows for analysis of movement quality as well as quantity, which has previously only been possible via self-report. Recently there has been a trend in using wearable sensors to measure mobility in individuals with PD at home [for review see (11)]. Detection of movements during functional mobility tasks appears accurate, and free-living assessment using wearable sensors is able to discriminate pathology better than testing in the laboratory (11).

The most common wearable sensors used to assess symptoms and motor complications of PD are accelerometers and angular rate gyroscopes (12). Accelerometers measure the accelerations of objects along sensor-fixed reference axes (12), while gyroscopes measure angular velocity around sensor-fixed reference axes (13). Sometimes accelerometers and gyroscopes are paired in an inertial measurement unit (IMU) (14); this combination of sensors can be fused to provide a stable estimate of sensor orientation (15, 16), as well as both linear and angular motion information. Additionally, surface electromyography (SEMG) uses electrodes on the skin to measure the electrical activity of muscle contraction. However, SEMG has mostly been used for short-term studies due to challenges with sensor placement repeatability. Only recently have products allowing long-term SEMG monitoring been introduced (17, 18).

### Algorithms for Identifying Movement Disturbances

Because wearable sensors produce large quantities of data that are not amenable to human interpretation, movement disturbances are usually studied through application of machine learning algorithms. Examples of machine learning techniques used in PD symptom classification include decision trees, neural networks (NNs), support vector machines (SVMs), linear discriminant analysis (LDA), Bayesian networks, and hidden Markov models (HMMs). Each of these methods, described briefly below, uses different methods for processing data and building up rules to detect signal features that correlate with the different categories to be classified.

A decision tree is a flowchart-like algorithm with nodes that represent a test on an attribute, branches that represent the outcomes of each test, and leaves that represent the classification (e.g., “tremor” or “freezing of gait”). The overall structure represents a set of rules that are exhaustive and mutually exclusive (19). One type of decision tree is called a Random Tree, which uses random processes to form the tree. In addition, a Random Forest algorithm can be made from a collection of Random Trees, where the output is the mean prediction of the individual trees (19).

Neural networks are used to classify data in a similar way to the neurons in a brain. They typically have multiple layers which perform different transformations on the inputs, and different weights are given to the layers. Although NNs typically perform well, a disadvantage to using NNs is that they are essentially “black boxes,” and there are no clear rules for how the input is turned into the output. Dynamic neural networks (DNNs) utilize feedback between “neurons” and layers, so that the weights are adjusted and they “learn” as they receive more data (20).

Support vector machines (SVMs) use non-linear boundaries between classification outputs; they transform the data into higher dimensions to find non-linear hyperplanes that divide the feature space. Dynamic support vector machines (DSVMs) incorporate discounted least squares (DLS), an error measure criterion that places more weight to recent training data points than distant training data points using dynamic parameters (21) to account for how patterns change over time (22). Linear discriminant analysis (LDA) uses linear combinations of features to construct classifiers that separate the categories as much as possible (23). Naïve Bayes is a form of probabilistic classifier, based on the Bayes theorem of independence among variables (19). Finally, a hidden Markov model is a dynamic learning structure that estimates a sequence of proposed states (such as normal, FOG, walking, tremor, etc.) from observed data, by estimating a probabilistic model of transitions among states and of observing certain measurements in each state. The state sequence through which the model passes to get to the output is hidden (22).

Each of the algorithms listed here has computational as well as functional strengths and weaknesses. To date, no system has been identified as the universally accepted and optimized algorithm for analysis of human movement.

### Summary of Prior Reviews

Previous review papers have summarized the use of wearable technologies to monitor PD but have not addressed the number, types, placement and data processing of sensors used to detect and quantify motor symptoms in a home setting. For example, Kubota et al. explored machine learning and how it might be used to address the symptoms of PD (24). Maetzler et al. focused on the comparison of individuals with PD to controls in clinical environments (25). Similarly, Godinho et al. focused on devices used to assess PD, but the studies reviewed did not take place in a home setting and did not focus on symptom detection (26). Del Din et al. reviewed studies in free-living settings, but focused on single sensor-based devices (11), while Hobert et al. covered ambulatory assessment in PD, but did not address symptom detection or severity assessment (27). None of these reviews have addressed the intersection of wearable technologies, machine learning, in-home monitoring, and symptom assessment in home or home-like settings during the performance of ADLs.

### Purpose of This Review

This paper summarizes and compares previously published research focused on monitoring the symptoms of individuals with PD in a home-like setting using wearable sensors, with a focus on performance of ADLs and instrumental activities of daily living (IADLs). ADLs require basic skills and focus on self-care abilities such as bathing, using the toilet, dressing/undressing, grooming, functional ambulation, and eating (28). IADLs require more advanced skills and consist of activities such as using the telephone, shopping, food preparation, housekeeping, laundry, using transportation, using medication, and handling finances (28). The included studies on symptom assessment during ADL and IADL performance include a wide range of specific tasks as well as continuous recording methods which gather data from all tasks undertaken during the recording period. We specifically address the number and types of sensors used, where the sensors were placed, and the type of symptoms that were quantified. Progress in monitoring tremor, freezing of gait, dyskinesias, bradykinesia, and hypokinesia during ADLs and IADLs are reviewed.

## Materials and Methods

A literature search was performed through the electronic databases PubMed/Medline and Google Scholar on multiple occasions through September 2018 using the search terms “Parkinson's disease”, “sensor”, and “activities daily living” as free text. The references from articles were scanned to identify additional studies, as well as articles cited by them. The abstracts of the articles found were read, and studies were included in the review if they involved objective monitoring of motor symptoms in patients with PD using wearable sensors, with a focus on studies done in a home or home-like setting. Articles were excluded if the methodology would only work in a laboratory setting.

## Results

From the literature search, 27 studies were found that met the criteria. Studies were found that measured tremor, bradykinesia, hypokinesia, dyskinesia, akinesia, and freezing of gait, with some studies containing more than one motor disorder (indicated with an ^*^ in Tables 1–4). The types of sensors used in the studies included accelerometers, SEMG, and gyroscopes, with some studies using a combination of sensors. Five studies measured tremor, five measured bradykinesia or hypokinesia, 13 measured dyskinesia or motor fluctuations, and ten measured akinesia or freezing of gait. Tables 1–4 summarize the results.

**Table 1:** Studies on tremor.

**Study**	**Location**	**Sensors**	**Protocol**	**Metrics**	**Algorithm**	**Results**
Hoff et al. (29)	Part 1: Lab	Part 1: 3 uni-axial accelerometers on one wrist	Part 1: seated posture; measured at rest and while performing motor tasks	Part 1: amplitude, dominant frequency, duration, bandwidth	Part 1: FTFT, detect tremor if longer than minimal duration (1.5 s) of dominant frequency, with limited bandwidth (1.0 Hz), and greater than minimal amplitude (0.01 g)	Part 1: Tremor vs. no tremor compared to specialists: SENS > 82%; SPEC > 93%
	Part 2: Home	Part 2: same as Pt 1; also 2 pairs uni-axial accelerometers (sternum and upper dominant leg)	Part 2: Measured for 24-h while keeping diary	Part 2: same as Pt 1; also duration of posture, duration of tremor, mean amplitude of tremor	Part 2: same as Pt 1; also determine standing vs. sitting based on gravitational vector	Part 2: Duration of tremor moderately correlated with UPDRS score for resting tremor (ρ = 0.66 standing, 0.77 sitting) Intensity of tremor correlated with resting tremor (ρ = 0.70 standing, 0.75 sitting)
^*^Roy et al. (30)	Simulated home	SEMG and tri-axial accelerometer over wrist extensors and tibialis anterior	4 h moving freely while videotaped	Energy, lag of first peak in autocorrelation of signal, ratio of height of first peak to height of peak at origin	DNN	Tremor vs. no tremor compared to specialists: SENS > 88%, SPEC > 91% Severity level of tremor on UPDRS compared to specialists: SENS > 95%, SPEC > 97%
^*^Cole et al. (31)	Simulated home	SEMG and tri-axial accelerometer over wrist extensor of dominant arm	4 h moving freely while videotaped	Energy, lag of first peak in autocorrelation of signal, ratio of height of first peak to height of peak at origin	DNN	Tremor vs. no tremor compared to specialists: SENS = 93%, SPEC = 95%
^*^Cole et al. (22)	Simulated home	SEMG and tri-axial accelerometer over symptomatic wrist extensor and symptomatic tibialis anterior	3–4 h moving freely while videotaped	Energy, lag of first peak in autocorrelation of signal, ratio of height of first peak to height of peak at origin	DNN, DSVM, and HMM for tremor detection; Bayesian maximum likelihood classifier for tremor severity classification	Tremor vs. no tremor compared to specialists: DNN: GER 6.2%, LER 0.28% DSVM: GER 7.2%, LER 0.41% HMM: GER 6.1%, LER 0.45% UPDRS tremor severity classification compared to specialists: SENS > 95%, SPEC > 95%
^*^Salarian et al. (32)	Hospital	Part 1: 3 uni-axial gyroscopes near wrist	Part 1: 45 min of 17 ADL while videotaped (DBS on and DBS off)	Dominant pole frequency and amplitude	Part 1: IIR filter with 3 s windows and autoregressive model. Tremor detected if frequency between 3.5 and 7.5 Hz and amplitude > 0.92. Tremor amplitude estimated from RMS angular velocity.	Part 1: Tremor vs. no tremor compared to specialists: SENS = 99.5%, SPEC = 94.2% Estimated tremor amplitude from roll axis showed high correlation (*r* = 0.87) to the UPDRS tremor subscore
		Part 2: 2 uni-axial gyroscopes (roll and pitch) near wrist	Part 2: 3–5 h moving freely			Part 2: Est. tremor amplitude: insensitive to window duration (*r* > 0.7 for window sizes 5–45 min)

*FTFT, fast time frequency transfer; ρ, Spearman rank correlation; DNN, dynamic neural network; DSVM, dynamic support vector machine; HMM, hidden Markov model; GER, global error rate; LER, local error rate; DBS, deep brain stimulation; IIR, infinite impulse response; RMS, root mean square; r, Pearson correlation; SENS, sensitivity; SPEC, specificity*;

* *, study addressing multiple motor disorders*.

**Table 2:** Studies on bradykinesia and/or hypokinesia.

**Study**	**Location**	**Sensors**	**Protocol**	**Metrics**	**Algorithm**	**Results**
Dunnewold et al. (33)	Home	Pairs of uni-axial accelerometers on sternum, upper leg, and wrist	24-h continuous recording	Bradykinesia: magnitude of acceleration for arm and leg; Hypokinesia: MIP (period with acceleration below a threshold) for hand and trunk	Discriminant analysis to determine thresholds, Multiple regression analysis for objective measures and UPDRS scores	Bradykinesia: mean arm and leg accelerations showed inverse relation with ipsilateral UPDRS motor score (R2 = 0.1, R2 = 0.45) Hypokinesia: arm MIP and trunk MIP lengthened with increasing ipsilateral UPDRS motor scores
^*^Klapper et al. (34)	Main room of a day program for PD	Tri-axial accelerometers near the wrists, ankles, and hip	2 subjects recorded for about 320 min each while videotaped	Absolute value of derivative of magnitude of acceleration and position and magnitude correlation between sensors	Classification trees and neural networks	Bradykinesia/ hypokinesia vs. no bradykinesia/ hypokinesia compared to neurologist: Neural network with c-index of 88.0–92.1% Classification tree with accuracies of 74.8–85.3%
^*^Patel et al. (35)	Lab	Tri-axial accelerometers on upper arms, forearms, supper thighs, and shins	Standardized clinical motor tasks (alternating hand movements, finger to nose, and heel tapping) while videotaped	Intensity (RMS), auto-covariance, dominant frequency, correlation features, and entropy	Clustering evaluation index to select features and linear discriminant classifier to predict performance of features	Best features for predicting clinical scores of bradykinesia on UPDRS were approximate entropy and intensity (RMS of acceleration) Optimal window length 6 s
^*^Salarian et al. (32)	Hospital	Part 1: 3 uni-axial gyroscopes near wrist	Part 1: 45 min of 17 ADL while videotaped (DBS on and DBS off)	Mobility of hand (RMS of angular velocity), range of rotation of hand (integration of angular velocity), activity of hand (percentage of time in window with movement)	Lowpass filter to remove tremor, considered only periods with instantaneous amplitude >5°/s as periods of movement	Part 1: significant correlation between mobility (*r* = 0.83) and range of rotation of hand (*r* = 0.70) from roll axis with UPDRS bradykinesia subscore
		Part 2: 2 uni-axial gyroscopes (roll and pitch) near wrist	Part 2: 3–5 h moving freely			Part 2: mobility showed highest correlation with UPDRS bradykinesia, insensitive to window duration (*r* > 0.74 for window sizes 5–45 min)
Samà et al. (36)	Home	Tri-axial accelerometer on waist near iliac crest	10–30 min of scripted ADL before and after antiparkinsonian medication while videotaped	Motion fluency (energy in the 0–10 Hz band) for strides in each walking bout	Gait detected from SVM, then motion fluency of strides compared to patient-dependent threshold; Severity of bradykinesia from ϵ-SVR model	Bradykinesia vs. no bradykinesia compared to specialists: SENS = 92.5%, SPEC = 89.1% High motion fluency correlation with UPDRS scores (*r* > 0.8) Low average errors in estimating bradykinesia severity on UPDRS (NRMSE = 14.75%)

*MIP, mean immobility period; SVM, support vector machine; ϵ-SVR, epsilon support vector regression; NRMSE, normalized root mean squared error; SENS, sensitivity; SPEC, specificity*;

* *, study addressing multiple motor disorders*.

**Table 3:** Studies on dyskinesia and/or motor fluctuations.

**Study**	**Location**	**Sensors**	**Protocol**	**Metrics**	**Algorithm**	**Results**
Hoff et al. (37)	Home	Uni-axial accelerometers: 2 on sternum (sagittal and coronal axes), 3 on the wrist (sagittal, coronal, and transverse axes), and 2 just above the knee of the most affected side (sagittal and coronal plane)	24-h continuous recording, and during each 30 min period, self-assessed presence of “on”, “off”, and “dyskinesias”	Bradykinesia: mean acceleration of arm; Hypokinesia: MIP of arm; Tremor: % of time with tremor; Dyskinesia: Mean acceleration of leg and MIP of trunk	Evaluated mean acceleration of arm, MIP, and mean tremor duration for “on” and “off” periods, and compared “on” and “off” using a Student *t*-test; Pearson correlation coefficients computed for dyskinesia measures and time spent with dyskinesia	Differences between “on” and “off” states not significant High correlation (**r** = 0.89) between dyskinesia objective measures and time spent with dyskinesias from self-assessment
Hoff et al. (38)	Lab	Uni-axial accelerometers: 2 on upper leg, 2 on wrist, 2 on trunk, and 2 on upper arm of the most affected side (coronal and sagittal planes for all)	Tasks 1–3: sitting in chair, counting forward, and spelling words backwards; Tasks 4–7: drinking from cup, putting on coat, buttoning, and walking	Mean amplitude of dominant frequency within the 1–4 Hz band (Amp 1–4) and 4–8 Hz band (Amp 4–8)	FTFT then computed Spearman correlation between mean amplitude of dominant frequency and m-AIMS score based on rating from clinical researchers	Tasks 1–3: Amp1–4 and Amp 4–8 from coronal wrist showed high correlation with m-AIMS (*r* > 0.80) Tasks 4–7: Variable correlations, but better results using sensors from body parts not involved in the task
Keijsers et al. (39)	Lab	Same as (38)	Same as (38)	38 parameters from the mean segment velocities (square root of sum of squares of derivatives of accelerometer signals) and cross-correlation between accelerometers	Neural networks using 38 input values and 16 input values	Higher Spearman correlations than using method from (38) Small mean error between neural network and m-AIMS score (on training data error around 0.5 for 16 inputs and on leave-one-out data error around 0.7 for 16 inputs)
Keijsers et al. (40)	Simulated home	Tri-axial accelerometers just below shoulders, halfway up upper legs, top of the sternum, and wrist of more affected side	2.5 h of approximately 35 scripted ADL while videotaped	92 features for each 1-min interval from derivative of accelerometer signal, power for frequencies in 1–3 Hz, power for frequencies above 3 Hz, and cross-correlation between accelerometers	Neural network to assess severity of dyskinesias (assessed as correct if within 0.5 of m-AIMS score)	Correctly classified dyskinesia/no dyskinesia compared to physician in 15-min intervals for 99.7% trunk, 93.7% arm, and 97% leg Correlation with m-AIMS score trunk *r* = 0.96, arm *r* = 0.88 and leg *r* = 0.92
^*^Klapper et al. (34)	Main room of a day program for PD	Tri-axial accelerometers near the wrists, ankles, and hip	2 subjects recorded for about 320 min each while videotaped	Absolute value of derivative of magnitude of acceleration and position and magnitude correlation between sensors	Classification trees and neural networks	Dyskinesia/no dyskinesia compared to neurologist: Neural network with c-index of 91.1–94.1% Classification trees with accuracies of 80.6–91.6%
^*^Patel et al. (35)	Lab	Tri-axial accelerometers on upper arms, forearms, upper thighs, and shins	Standardized clinical motor tasks (alternating hand movements, finger to nose, and finger tapping) while videotaped	Signals from lower extremity body parts (intensity (RMS), auto-covariance, dominant frequency, correlation features, and entropy)	Clustering evaluation index to select features and linear discriminant classifier to predict performance of features	Best features for predicting clinical scores of dyskinesia were entropy and intensity Worst features were correlation features Optimal window length 5 s
Samà et al. (41)	Lab while videotaped and outdoors with trained observer	Tri-axial accelerometer on belt	Lab: walking straight line, over inclined plane, carrying heavy object, setting table, and stairs; Outdoors: walking for at least 15 min	Power spectrum of acceleration	Dyskinesia detected when power spectrum between 1 and 4 Hz was above a threshold, and power spectrum below 20 Hz was below a threshold for window length of 6 s	Dyskinesia vs. no dyskinesia compared to specialist: SENS = 89%, SPEC = 78%, PPV = 98%, NPV = 87%
Rodríguez-Molinero et al. (42)	Home	Tri-axial accelerometer on belt	3–5 h performing habitual activities with observer trained in motor symptoms recognition	Motion fluency (energy in the 0.1–10 Hz band) for strides in each walking bout	Gait detected from SVM, then motion fluency compared to patient-dependent threshold (found through SVM with linear kernel)	Detecting “off” vs. “on” compared to specialist: Using all detected walking segments: SENS = 91%, SPEC = 90%, PPV = 80%, NPV = 94%. Using only walking segments containing 10 or more strides: SENS = 96%, SPEC = 94%, PPV = 90%, NPV = 98%
^*^Cole et al. (31)	Simulated home	SEMG and tri-axial accelerometer over wrist extensor of dominant arm	4 h moving freely while videotaped	Energy, lag of first peak in autocorrelation of signal, ratio of height of first peak to height of peak at origin	DNN	Dyskinesia vs. no dyskinesia compared to specialist: SENS = 91%, SPEC = 93%
^*^Roy et al. (30)	Simulated home	SEMG and tri-axial accelerometer over wrist extensors and tibialis anterior	4 h moving freely while videotaped	Energy, lag of first peak in autocorrelation of signal, ratio of height of first peak to height of peak at origin	DNN	Dyskinesia vs. no dyskinesia (from arm sensor) compared to specialist: SENS = 90%, SPEC = 91% Severity of dyskinesias compared to m-AIMS score: SENS > 91%, SPEC> 94%
^*^Cole et al. (22)	Simulated home	SEMG and tri-axial accelerometer over symptomatic wrist extensor and symptomatic tibialis anterior	3–4 h moving freely while videotaped	Only used accelerometer features: energy, lag of first peak in autocorrelation of signal, ratio of height of first peak to height of peak at origin	DNN, DSVM, and HMM for dyskinesia detection; Bayesian maximum likelihood classifier for dyskinesia severity level detection	Dyskinesia vs. no dyskinesia compared to specialist: DNN: GER 8.8%, LER 4.8% DSVM: GER 9.1%, LER 6.4% HMM: GER 12.3%, LER 4.2% Dyskinesia severity level compared to m-AIMS score: SENS and SPEC > 91%
Pulliam et al. (43)	Lab	IMUs on dorsum of wrists, anterior surfaces of thighs, and lateral aspects of ankles	Scripted ADL once per hour for 3 h while videotaped	18 time and frequency domain features from acceleration and angular velocity	Linear regression model	All sensors: high correlation with m-AIMS score (*R* = 0.86) with NRMSE 7.4% Wrist and ankle sensors: high correlation with total m-AIMS score (*R* = 0.77) with NRMSE = 9.4% Wrist and thigh sensors: high correlation with m-AIMS score (*R* = 0.74) and NRMSE = 9.8%
Rodríguez-Molinero et al. (44)	Home	Tri-axial accelerometer on waist	Recorded for 1–3 days while keeping a diary of “on” and “off” periods every 30 min	Bradykinesia: movement fluidity (power spectrum between 0 and 10 Hz); Dyskinesia: power spectrum between 1 and 4 Hz	Gait detected from SVM; Bradykinesia if movement fluidity above threshold; Dyskinesia if power 1–4 Hz above threshold; “On” if dyskinesia but no bradykinesia; “Off” if bradykinesia but no dyskinesia	Detecting “off” vs. “on” periods compared to self-assessment: PPV = 92%, NPV = 94%, overall classification accuracy = 92%

*MIP, mean immobility period; r, Pearson correlation coefficient; FTFT, fast time frequency transfer; RMS, root mean squared; SVM, support vector machine; DNN, dynamic neural network; DSVM, dynamic support vector machine; HMM, hidden Markov model; GER, global error rate; LER, local error rate; NRMSE, normalized root mean squared error; SENS, sensitivity; SPEC, specificity; PPV, positive predictive value; NPV, negative predictive value*;

* *, study addressing multiple motor disorders*.

**Table 4:** Studies on akinesia and/or freezing of gait.

**Study**	**Location**	**Sensors**	**Protocol**	**Metrics**	**Algorithm**	**Results**
Morris et al. (45)	Lab	Uni-axial (vertical) accelerometer on lateral leg near ankle	Timed up and go test while videotaped	Power spectrum of vertical acceleration signal	iFOG: FOG if ratio of power in freeze band (3–8 Hz) to power in locomotor band (0.5–3 Hz) greater than threshold (2) for 10 s windows	Correlation 0.78 for number of FOG events (compared to average of clinicians) Correlation 0.93 for percent time frozen (compared to average of clinicians)
Moore et al. (46)	Lab	IMU on left leg just superior to the ankle	Walking around corridors, through doorways, and around obstacles (during “off” state then periodically repeated over a 90-min period after taking medication)	Power spectrum of vertical leg acceleration	FOG identified when normalized freeze index (ratio of power in 3–8 Hz to power in 0.5–3 Hz) above a threshold for 6 s sliding windows	FOG vs. no FOG compared to specialist: Using global threshold (2.3): detected 78.3% of FOG events with 20% FP rate Using individual thresholds (2.3–4.4): detected 89.1% of FOG events with 10% FP rate
Bächlin et al. (47)	Lab	Tri-axial accelerometers just above the ankle, just above the knee, and lower back on a belt	3 basic walking tasks (straight walking, turns, and ADL such as carrying glass of water) while videotaped	Energy in locomotor band (0.5–3 Hz), energy in freeze band (3–8 Hz), and complete energy (0.5–8 Hz) from ankle vertical accelerometer	If complete energy is above power-threshold, FI calculated (ratio of energy in freeze band to energy in locomotor band) and FOG detected when FI exceeds freeze-threshold for 4 s windows	FOG vs. no FOG compared to physiotherapists: Online detection using global parameters SENS = 73%, SPEC = 81.6% Using smooth vs. saccadic group parameters SENS = 85.9%, SPEC = 90.4% Using individual parameters SENS = 88.6%, SPEC = 92.8%
Cole et al. (48)	Simulated home	Tri-axial accelerometers on forearm, thigh, and shin; SEMG on shin	Unscripted and unconstrained ADL while videotaped	8 features from accelerometers on forearm and shin, 3 features from SEMG	Linear classifier (using gravitational component of acceleration) to detect if upright, then if upright >5 s, DNN to detect FOG	FOG vs. no FOG compared to specialist: SENS = 83%, SPEC = 97% By ignoring FOG events <1 s and >8 s away from other FOG events: SENS = 82%, SPEC = 99%
Tripoliti et al. (19)	Lab	Tri-axial accelerometers near ankles, wrists; IMUs on chest, and waist	Scripted ADL (approximately 18 min, repeated in “off” and “on” states) while videotaped	Entropy from all sensors	Naïve Bayes, Random Forests, Decision Trees, and Random Tree	Detecting FOG vs. no FOG compared to clinician: Best result was Random Forests with SENS = 81.9%, SPEC = 98.7%
Tay et al. (49)	Lab	IMUs on ankles and back of neck	Timed up and go test and walking	Body posture from accelerometers, swing phase (peak in z-axis gyroscope), toe off and heel strike (troughs in z-axis gyroscope), and stride time	FOG defined when a certain amount of time passed (based on average stride time) and no forward movement (based on angular velocity threshold)	Didn't capture enough FOG events to make a correlation, but results were consistent with findings of loss of stride length and accelerated cadence at onset of FOG
Mazilu et al. (50)	Lab	IMUs on wrists and ankles	Walking tasks in “on” state to provoke FOG (turns, Figure 8, cognitive dual tasks, crowded hallways, elevator) while videotaped	Acceleration and angular velocity magnitudes (mean and STD), power spectrum of acceleration	Decision tree	FOG vs. no FOG compared to clinician using wrist: Subject dependent hit rate = 85%, and SPEC = 80% Subject independent hit rate = 90%, SPEC = 66% 40% more FPs than ankle and higher latency of 1.15 s compared to 0.6 s for ankle
Azevedo Coste et al. (51)	Lab	IMU on shank	Walking along a 10 m corridor during dual tasks while videotaped	Stride length and cadence (from segmentation of gait data into strides)	FOG detected if FOGC (based on cadence and stride length) is above a threshold	FOGC: missed only 4 of 26 FOG events compared to clinician FI method (46): missed 9 of 26 FOG events compared to clinician
Rodríguez-Martín et al. (52)	Home	Tri-axial accelerometer on waist	20 min of scripted activities (walking around home, doorways, walk outside, and dual task) before and after medication (after also did ADL with short and fast movement) while videotaped	55 features (such as means, increment between windows, correlation between axes, and spectral information) from acceleration data	SVM	FOG vs. no FOG compared to specialist: Generic model SENS = 74.7%, SPEC = 79% Personalized model SENS = 80.1%, SPEC = 88.1%
Prateek et al. (53)	Lab	IMU on heel	5 gait tasks (walking backwards, stepping over a block, Figure 8, narrow path, and in-place 180° turns)	Acceleration and angular velocity signals, foot speed from IMU data	Filtered out gait patterns that are not ZVEI or TREI using accelerometer signal Distinguished ZVEI from TREI detected above using accelerometer and gyroscope signals Used a point-process filtering module by combining speed of foot from foot-mounted IMU with the detected TREI via a conditional intensity function to compute pFOG	FOG vs. no FOG compared to gait analysis experts: 81.03% detection accuracy using a tuned participant-specific parameter 72.41% detection accuracy using a fixed value parameter Three-fold decrease in false-alarm rate compared to FI method

*iFOG, index of FOG; FI, freezing index; DNN, dynamic neural network; STD, standard deviation; FOGC, FOG criterion; SVM, support vector machine; SENS, sensitivity; SPEC, specificity; FP, false positive; ZVEI, zero-velocity event intervals; TREI, trembling event intervals; pFOG, probability of FOG*.

Tables 1–4 are organized according to PD symptoms to provide a convenient reference on how different symptoms of PD can be measured with various sensor technologies. However, each symptom can be addressed with multiple different sensors, and a chosen sensor system can be used with different algorithms to report on multiple symptoms. Therefore, to help the reader determine which sensor or combination of sensors is most appropriate for measuring multiple symptoms of interest, the text of the results section is organized according to sensor type, and summarizes the symptoms each sensor has successfully measured.

### Studies Using Gyroscopes

Gyroscopes were not commonly used alone to assess PD symptoms in home-like settings. Only one paper was found that used gyroscopes and the only motor symptoms quantified were tremor and bradykinesia.

Salarian et al. used sensors consisting of uni-axial gyroscopes just above the wrists to detect the presence of tremor, quantify tremor amplitude and assess bradykinesia (32). The first part of the study involved 17 typical ADL and IADL tasks that could be completed in a hospital environment (e.g., sitting, walking, writing, eating and drinking, brushing teeth, combing hair); a second test collected data for 5 h continuously using 2 gyroscopes (roll and pitch) on each forearm above the wrist. In both tests, the estimated tremor amplitude from root mean square (RMS) of the roll axis showed high correlation to the UPDRS tremor subscore, and mobility of the hand (RMS angular velocity) correlated with the UPDRS bradykinesia subscore. Range of rotation of the hand (integration of angular velocity) correlated with the UPDRS bradykinesia subscore only in the shorter ADL/IADL test. The authors also suggested that the activity of the hand (percentage of time in a window with movement >5°/s) would be a good estimator of hypokinesia.

### Studies Using Accelerometers

Accelerometers were the most common instrumentation used to assess PD symptoms with 26 total papers found that used accelerometers. Of these 26 papers, 15 used accelerometers in isolation. The symptoms addressed included tremor, bradykinesia, hypokinesia, dyskinesia, motor fluctuations, and freezing of gait.

#### Tremor

Hoff et al. (29) used three uni-axial wrist-mounted accelerometers and two pairs of uni-axial body mounted accelerometers (one pair mounted radially on the sternum, the second pair mounted radially on the upper dominant leg) to detect the presence and duration of tremor. Data were continuously recorded over a 24-h period while the individual was at home. During the 24-h continuous recording, it was found that the duration and intensity of tremor correlated with the UPDRS score for resting tremor.

#### Bradykinesia, Hypokinesia, Dyskinesia and Motor Fluctuations

Dunnewold et al. (33) used pairs of uni-axial accelerometers mounted perpendicular to each other radially on the sternum, upper leg (most affected side), and wrist (most affected side) during 24-h of continuously recorded movements. For bradykinesia, the mean arm acceleration and the mean leg acceleration in the upright position showed a modest inverse relation with the UPDRS motor score of the most affected side. For hypokinesia, the arm and trunk mean immobility periods (MIP), periods without acceleration above a threshold, lengthened with increasing ipsilateral UPDRS motor scores, but the change was not significant (33).

Samà et al. (36) used a single waist-worn triaxial accelerometer to detect presence and severity of bradykinesia. Individuals with PD performed a set of scripted ADL tasks at home (such as walking around their home, carrying a glass of water, and a freezing-of-gait provocation test) both before and after taking their prescribed antiparkinsonian medication. For the bradykinesia detection method, first strides were identified through a support vector machine (SVM) model which is based on signal power in frequency bands. Then motion fluency (signal power in the 0–10 Hz band) for strides in each walking bout was compared to a patient-dependent threshold. Motion fluency values were also used in an epsilon support vector regression (ϵ-SVR) model to detect severity of bradykinesia based on UPDRS scores. Bradykinesia vs. no bradykinesia was detected (compared to a specialist) with very high sensitivity and specificity, and low error was found between the bradykinesia severity estimation compared to the UPDRS-III item 24 (body bradykinesia and hypokinesia) (36).

Accelerometry was also used for detecting motor fluctuations and dyskinesias. Klapper et al. (34) used five tri-axial accelerometers (on the dorsum of each arm just proximal to the wrist, on each leg just proximal to the lateral aspect of the ankle, and on the hip attached to the patient's belt) to detect bradykinesia, hypokinesia, and dyskinesias while the individuals went about their typical daily functions in the main room of a day program for persons with PD (34). The authors used classification trees and neural networks (NNs) to detect bradykinetic/hypokinetic states vs. not bradykinetic/hypokinetic states compared to dichotomized scores from the neurologist who observed the participants for the duration of the recording period. NNs performed better in detecting both bradykinetic/hypokinetic states vs. not bradykinetic/hypokinetic states and dyskinetic states vs. non-dyskinetic states (34). Patel et al. (35) used tri-axial accelerometers on the upper arms, forearms, upper thighs, and shins to predict clinical scores of bradykinesia and dyskinesia on the UPDRS during standardized clinical motor tasks such as finger tapping, alternating hand movements, and heel tapping (35). Although their protocol included clinical motor tasks rather than ADLs, this study was included because the system would be able to be used in the home. They found that the best features to predict bradykinesia scores were approximate entropy and intensity, as well as correlation and frequency features (35). Approximate entropy was defined as a measure of signal complexity, where a lower value has many repetitive patterns, and a high value indicates a complex signal. Intensity was measured as the RMS value of the accelerometer signal. Correlation features referred to coordination between body segments on the left and right side and proximal and distal segments, and features included magnitude, delay, and similarity. Dyskinesia was examined by choosing signals from lower extremity body segments during tasks requiring fine motor control of the upper extremities (such as alternating hand movements and finger tapping). Entropy and intensity were again found to be the best features to predict dyskinesia clinical scores (35).

Accelerometers were also used for detection of levodopa medication state where “off” state was defined as the period of time where hypokinesia, bradykinesia, and tremor occur, while dyskinesias occur in the “on” state. Hoff et al. (37) used two uni-axial accelerometers on the sternum (sagittal and coronal), three uni-axial accelerometers on the wrist (sagittal, coronal, and transverse), and two uni-axial accelerometers on the upper leg just above the knee of the most affected side (sagittal and coronal) during a 24-hour period (37). Mean acceleration of the arm, mean immobility period, and mean tremor duration [detected from the method in (29)] were compared to participants self-assessed “on” state, “off” state, and presence of dyskinesias every 30 min. Additionally, mean acceleration of the leg and mean immobility period of the trunk were used for objective measures of dyskinesias. Differences between the “on” and “off” states were not statistically significant. Overall, they concluded their method was not suitable for automated “on”/ “off” detection in individual patients, and theorized that NNs would perform better (37). However, they did find a high correlation between dyskinesia objective measures and time spent with dyskinesias from self-assessment (37).

Levodopa-induced dyskinesias (LID) during performance of ADL tasks were also assessed via accelerometry (38). Hoff et al. used four pairs of uni-axial (coronal and sagittal planes) accelerometers (two on the upper leg, two on the wrist, two on the trunk, and two on the upper arm of the most affected side) to investigate movement characteristics of LID in two frequency bands (1–4 Hz and 4–8 Hz) (38). Voluntary movements occur in the 1–4 Hz band, so the correlation was variable in that band (38). Interestingly, correlation between the objective measures and the m-AIMS score was better when using sensor data from body segments not performing the ADL task (38). For example, they found high correlation from the leg sensors during sitting and standing tasks that require fine motor skills. Keijsers et al. used the same data but with NNs to try to better differentiate between voluntary movements and LID and to better predict the severity of LID (39). They found low mean errors in predicting the m-AIMS score, and the authors concluded that NNs did a good job of distinguishing LID from voluntary movements (39).

Keijsers et al. also used data from tri-axial accelerometers on the upper arms (just below the shoulders), halfway up the thigh, the wrist of the most dyskinetic side, and the top of the sternum to detect the presence and severity of dyskinesia on the m-AIMS scale in a home-like setting using NNs (40). Subjects performed 2.5 h of approximately 35 scripted ADL and IADL tasks while wearing the sensors and being videotaped (40). Tasks included walking, putting on a coat, making coffee, preparing lunch, and eating. The NN correctly classified whether there were dyskinesias or no dyskinesias with high accuracy and had high correlation with the m-AIMS score rated by physicians (40).

In an attempt to use fewer sensors for online monitoring, Samà et al. used a single tri-axial accelerometer worn on a belt to detect presence of dyskinesias while patients performed activities in a laboratory (such as walking in a straight line, walking over an inclined plane, carrying a heavy object, setting a table, and going up and down stairs while videotaped) and while walking outdoors for at least 15 min with a trained observer (41). Dyskinesia was detected by analyzing the spectrum of the accelerometer signals. They found high accuracy in detecting dyskinetic events vs. non-dyskinetic events as compared to the trained observer (41). In a follow-up study, a single tri-axial accelerometer was attached to the waist to detect “on”/“off” medication states (42). Data were recorded for 3–5 h while the participants performed their normal routine in their homes while accompanied by a trained rater. They found high accuracy with an SVM compared to the trained observer, and even greater results when only considering walking segments with 10 or more strides (42).

Optimal detection of motor fluctuations were also examined by Rodríguez-Molinero et al. (44) using a waist-worn triaxial accelerometer. Participants wore the sensor for a variable number of daytime hours for 1–3 consecutive days while simultaneously recording whether they were in an “on” or “off” state in a diary every 30 min. Based on the accelerometry readings, the algorithm output either presence or absence of bradykinesia plus presence or absence of dyskinesia every 10 min based on a similar methodology to the Hoff et al. study (37). They found the algorithm output was accurate compared to the patient's diary (44).

#### Freezing of Gait

Accelerometers have been commonly used to address freezing of gait. Morris et al. (45) used uni-axial (vertical) accelerometers on the lateral aspect of the legs just superior to the ankles to assess FOG episodes. Although this study did not include an ADL or home component, it was included because it focused on the comparison of objective measures of FOG from frequency characteristics of acceleration and clinical measures of FOG. Patients were assessed in the “off” state during performance of timed up-and-go (TUG) tasks, and the frequency and duration of FOG episodes were rated by ten clinicians via video. An index of freezing of gait (iFOG) was defined based on frequency characteristics of the vertical accelerometer data [ratio of signal power in the freeze band (3–8 Hz) to signal power in the locomotor band (0.5–3 Hz)]. FOG was detected if the iFOG was above a threshold for 10 s windows. Strong agreement was found between their algorithm and the mean of the scores from the ten clinicians (45).

Bächlin et al. focused on minimizing the complexity of the algorithm for use in online detection of FOG (47). A tri-axial accelerometer just superior to the ankle was used for online detection during three basic walking tasks including straight walking, turns, and during ADL tasks such as carrying a glass of water (47). Using a global threshold set to identify FOG vs. no FOG in real-time, they found acceptable sensitivity and specificity compared to video analysis by physiotherapists (47). They found even greater results when separating subjects into two walking style groups (smooth and saccadic) based on their freeze-threshold values. Finally, the optimal performance was found using individual thresholds for each subject.

Rodríguez-Martín et al. used a single waist-worn tri-axial accelerometer to detect FOG at home during scripted activities before and after taking medication (52). In addition, following medication a false positive protocol activity was performed which included short and fast movements similar in frequency content to FOG. FOG vs. no-FOG was classified using an SVM model compared to video rating by an experienced clinician, and acceptable results were found using a generic model. Better results were found using patient-specific models (52).

### Accelerometry Combined With Surface Electromyography

There were 4 papers found that used accelerometers in combination with surface electromyography (SEMG) to provide a more complete view of the recorded movements and the related muscle activity. Of these 4 papers, 3 used the hybrid sensors to detect tremor and dyskinesia and 1 used the sensors to detect freezing of gait.

Roy et al. used four hybrid sensors consisting of SEMG and triaxial accelerometers above both wrist extensor muscles and both tibialis anterior muscles in the shin (30) for 4 h of recording in an apartment-like setting performing unscripted and unconstrained activities. Tremor and dyskinesias were detected using separate DNNs, designed to learn how features of the movement change over time. The fully developed DNNs were able to detect presence and absence of tremor and dyskinesia from the upper body sensors and identify the clinical severity level compared to video analysis by specialists (30).

While Roy et al. used four sensors, Cole et al. used a single hybrid SEMG and a tri-axial accelerometer sensor attached near the origin of the wrist extensor muscle of the dominant arm to detect tremor (31). Data were recorded during 4 h of unscripted and unconstrained simulated daily activities, such as washing dishes, setting the table, and making a bed, in an apartment-like environment. Using DNNs, they were able to detect tremor vs. no tremor compared to the annotated video (31). They also successfully trained DNNs to detect dyskinesia (31). Building on this work, Cole et al. used two hybrid SEMG and triaxial accelerometer sensors, one on the origin of the wrist extensor muscle of the more symptomatic arm and one on the tibialis anterior muscle of the shin of the more symptomatic leg to detect tremor and compared the performance of different machine learning algorithms. Tremor was detected using three different dynamic machine learning structures: hidden Markov models (HMMs), dynamic neural networks (DNNs), and dynamic support vector machines (DSVMs) (22). The researchers also estimated tremor severity level on the UPDRS using a Bayesian maximum likelihood classifier on high-pass energy from the accelerometer signal. The researchers found a slight advantage to using the DNN algorithm to detect tremor vs. no tremor, although all methods were similarly effective. All algorithms performed well in estimating the tremor severity level also, with a slight advantage to using the DNN algorithm (22).

Cole et al. also used a set of combined sensors to detect FOG (48). Tri-axial accelerometers were placed on one forearm, thigh, and shin and an SEMG sensor was placed on the shin of patients and control subjects during unscripted and unconstrained ADLs in an apartment-like setting. If the subject was detected to be upright for five or more consecutive seconds, a DNN was applied to detect whether FOG occurred. The DNN was trained and tested on different datasets, and was found to have high sensitivity and specificity for detecting FOG vs. no FOG compared to specialists' ratings (48). Since false positives could be costly in a real system (e.g., by leading patients to undergo unnecessary treatment), they ignored FOG detections that were <1 s in length or isolated more than 8 s away from others, and improved their specificity and sensitivity (48).

### Accelerometers and Gyroscopes (Inertial Measurement Units)

Combining tri-axial accelerometers and gyroscopes into inertial measurement units (IMUs) is another common type of instrumentation, which exploits information in both linear and angular motion, and in both velocity and acceleration. There were 7 papers found that used accelerometers in combination with gyroscopes to provide a more complete view of the recorded movements. Of these 7 papers, 1 used the sensors to detect dyskinesia and 6 used the sensors to detect freezing of gait.

Pulliam et al. used IMUs on the wrists, thighs, and ankles of patients during scripted ADL tasks (such as drinking, dressing, buttoning a coat, combing hair, and cutting food) to examine the accuracy in predicting the total m-AIMS score using the sensor array (43). Linear regression models were trained to output m-AIMS severity scores, and they found high correlation between the average clinician total m-AIMS score and the model score for the 6-sensor array (43). They also tested configurations with just 2 sensors, to make the system more realistic for a home setting and still found acceptable results, suggesting reasonable accuracy using only two sensors (43).

Moore et al. used IMUs attached to the left leg just superior to the ankle to detect FOG during walking (46). The researchers defined a freeze index (FI) as the ratio of power in the freeze band (3–8 Hz) to power in the locomotor band (0.5–3 Hz) from the vertical accelerometer signal for 6-s sliding windows. The locomotor band was determined based on the frequency characteristics of the gait of the 11 subjects. FOG was identified when the FI was above a threshold. Using a global threshold, they were able to detect FOG events compared to specialists, with even greater results after establishing individual thresholds (46).

Tripoliti et al. used a combination of four stand-alone tri-axial accelerometers on the legs (near ankles) and wrists and two IMUs on the chest and waist to detect FOG during commonly performed tasks (19). Freezing events were identified by computing signal entropy within a 1-s sliding window. The researchers used the entropy estimates in four classification algorithms: Naïve Bayes, Random Forests, Decision Trees, and Random Tree. Best results were found using all sensors and the Random Forests algorithm (19).

Tay et al. used IMU's attached to the ankles and back to demonstrate feasibility of detecting FOG based on gait parameters (49). Accelerometer signals were used to monitor body posture during standardized tasks such as the timed up and go, 10 m walk, and free non-timed walk. Gyroscope signals were used to detect transitions from swing to stance phase on both legs (49). Freezing was defined when a certain amount of time passed, and no forward movement was detected. Average stride time and threshold levels were constantly updated to adapt to the patient's gait and helped determine the acceptable timing to wait for the next gait phase (49). Too few FOG events were captured to examine correlations between subjective and calculated events, but results were consistent with findings of loss of stride length and accelerated cadence at the onset of freezing. Similarly, Azevedo Coste et al. (51) detected FOG using gait parameters such as stride length and cadence based on the hypothesis that before freezing, cadence should increase and stride length should decrease. They created a complementary index freezing of gait criterion (FOGC) using continuous evaluation of cadence and stride length (51). Gait data from walking along a 10 meter corridor during several dual tasks was segmented using gyroscope data from an IMU on the shank in the sagittal plane, with the FOGC threshold adjusted for each patient (51). They were able to detect FOG events that were missed when using the standard freezing index (FI) method of Moore (46), however, the authors did note the drawback that the FOGC can only detect freezing during the gait cycle (51).

Mazilu et al. (50) compared FOG detection through wrist and ankle-worn sensors, because wrist placement is more likely than other locations to be accepted by elderly users. Using decision trees, the best wrist location (non-dominant wrist) had a similar hit rate compared to the best ankle location (dominant leg), but it had 40% more falsely detected events and a higher latency (50). The authors found that the best features to use for detection were participant specific (50). They also found that performing actions and gestures with the wrist increased false positives. However, they suggested that high false positive rates may not be critical for intervention applications because having fewer missed events is favorable to high precision because of the risk of falls with FOG.

Finally, Prateek et al. (53) developed a method using an IMU strapped to the heel region of the foot based on clinical observations that FOG patterns include trembling in the lower extremities and no movement of the limbs and trunk. Individuals with PD were tested while performing five gait tasks designed to trigger FOG (53). The first part of the algorithm filtered out events that were not considered zero-velocity event intervals (ZVEI) or trembling event intervals (TREI) using the accelerometer signal. The second part of the algorithm distinguished ZVEI from TREI using the gyroscope signal. Lastly, they used a point-process filtering module to compute the probability of FOG (pFOG) for the TREI events using information about the speed of the foot from the foot-mounted IMU (53). They compared their algorithm to a previous method and showed either an improvement or equivalent performance in detecting different types of FOG using a participant-specific tunable parameter. Use of a fixed value based on the average across all FOG participants showed better accuracy than the previously established FI method and a more than 3-fold decrease in false positives (53).

## Discussion

Monitoring of motor symptoms during activities of daily living (ADLs) in a home setting requires a system that is easily donned and doffed, unobtrusive and unrestrictive, contains as few separate sensor units as possible, allows for remote access to the data and can be updated and managed remotely allowing for a low-level of technical expertise by the end user. For individuals with PD, in-home monitors must also be designed to address motor symptoms specific to this population. In this review, sensors for measuring the presence, amplitude, duration, and intensity of each of the primary motor symptoms identified for individuals with PD has been examined and individual sensors or combination of sensors and processing algorithm(s) have been identified as more and less sensitive and specific. However, no single study was able to capture all the motor disturbances common in PD utilizing a sensor array. Future research should work toward a sensor system that will allow for remote clinical management of an individual's PD symptoms, progression, and possible disease related complications such as medication fluctuations and motor freezing.

The selection of an ideal sensor or sensor system for quantification of ADLs and IADLs is complex. For example, just the question of optimal anatomical location and number of sensors presents a unique challenge in a population of individuals with heterogeneous symptom presentation, unilateral motor symptom onset and motor fluctuations. The reviewed studies indicate that, depending on the specific motor symptom that is being assessed, sensor placement can vary significantly. For example, individuals with PD may only have a tremor present on one side of the body, thus necessitating a sensor on the tremor-dominated limb. However, brady- and hypokinesia are best detected in the limb that is actively involved in the given ADL task and dyskinesias detection is best done with sensors attached to regions of the body not utilized during the task. Finally, freezing of gait (FOG) is best detected with sensors on the dominant leg. These conflicting and confounding factors increase the difficulty in finding or developing a sensor array that can optimally address all relevant motor symptoms in a fashion useful for field-based data collection.

One means of maximizing the usefulness of the collected data from any one sensor or group of sensors is to process the data using adaptive algorithms that address the complexity and improve the accuracy of symptom detection. Neural networks or dynamic neural networks allow for processing of the data within the context of either prior collected data or data from the individuals themselves. A dynamic neural network is a more complex processing technique that could be used to improve the performance of a system with fewer sensors by capturing non-linear and complex relationships between features. Dynamic neural networks “learn” as the data are collected and therefore become more accurate and informative as the study progresses. Based on the reviewed papers, DNNs appear to provide optimized accuracy across tremor, bradykinesia, hypokinesia, and dyskinesia. DNNs showed superior performance in detection of tremor and dyskinesia as compared to DSVMs and HMMs (22) and NNs showed superior performance in detection of bradykinesia/hypokinesia vs. no bradykinesia/hypokinesia and dyskinesia vs. no dyskinesia as compared to classification trees (34). DNNs were also able to be trained in detection and assessment of freezing of gait (48).

An important consideration in the selection of both sensors and adaptive algorithms is the identification of false positive signals. Two studies brought up seemingly contradictory ideas about risk management in selecting the thresholds for FOG detection. One argued that false positive detections should be carefully avoided because these could influence patient care (48); whereas the other argued that false positives were largely acceptable because they lower the false negative rate and therefore improve the performance of a real-time intervention (50). These ideas are both valid, and the discrepancy lies in the context. The main risk of allowing false positives is in the diagnosis phase: individuals might be mistakenly classified as having FOG when they do not truly have FOG or having FOG of greater severity than they truly have. This mistake would lead to severe errors in healthcare such as treatment when none is needed or more aggressive treatment than needed. The main benefit of allowing false positives (or more precisely, of minimizing false negatives) is in the intervention phase: an individual experiencing FOG might be left “frozen” if the FOG is not detected (a false negative), yielding poor performance of the intervention. But applying an intervention when no FOG occurs is likely to be no worse than a mild annoyance. These examples illustrate the importance of considering the reasons for a detector and consequences of each type of failure when determining thresholds for any symptom detection. Use and optimization of DNNs would allow for clinicians to consider the use of the monitoring system and “train” the algorithms in the appropriate manner.

Determination of the optimal sensor type was largely based on the type of symptom being evaluated. However, accelerometers appear to be successful in the detection of tremor (29), bradykinesia/hypokinesia (33, 34, 36), dyskinesia (34, 38–41), motor complications (42, 44), and freezing of gait (45, 47, 52). The use of IMUs improved the detection accuracy for freezing of gait (19, 51, 53). Unfortunately, the optimal placement of the specific sensors varied greatly based upon the motor symptom being detected. It is also worth noting that in all cases the location of the sensor must allow for free and untethered use of the limb during the performance of ADL/IADL tasks.

Most tremor detection and quantification systems used accelerometer frequency features sensitive to the characteristics of resting tremor. Using just accelerometers, sensitivity above 80% and specificity above 90% was found (29). Combining accelerometer features with SEMG features and the use of neural networks increased sensitivity (30, 31). However, SEMG sensors are not ideal for long-term monitoring of symptoms at home. For best results, the electrode needs to be precisely placed over the muscle being measured, which individuals with PD may not be able to do on their own and the electrode may lose electrical contact throughout the course of the day. Future studies should consider the use of IMUs, which combine accelerometers and gyroscopes, for detecting tremor. This technique has been used repeatedly in laboratory-based studies with an eye toward in-home use (54, 55). It is likely that a small tremor detection system utilizing only accelerometers and/or gyroscopes could be implemented on the wrists, possibly in the form of a smart watch, and achieve excellent results using neural networks during unscripted and unconstrained activities in the home.

The most successful detection of bradykinesia and hypokinesia used accelerometers coupled with machine learning techniques to analyze the data. Studies that used threshold-based methods showed only modest correlations with UPDRS scores for bradykinesia (32, 33). Using neural networks or support vector regression models, the presence of bradykinesia was detected at rates above 88%, with the best results from neural networks (34, 36). Bradykinesia during gait can be detected using a single tri-axial accelerometer on the waist (36); however, during ADLs best results were found using sensors attached to body parts involved in the task (33). This dichotomy is unfortunate because it suggests bradykinesia may require multiple sensors; this inconvenience may severely limit its utility in practice. New approaches may be needed to retain high classification accuracy while minimizing the number of sensors required. Hypokinesia was best classified using metrics such as immobility periods (33) and low activity measures (32). Therefore, the promising single-sensor methods that utilize gait measurements would likely not be useful for hypokinesia, and sensors attached to body parts involved in the tasks being performed throughout the day may be needed.

Detecting dyskinesia during ADLs is particularly challenging because voluntary movement occurs in the same frequency band as dyskinetic movement. It appears that dyskinesia is best detected using sensors attached to body parts not involved in the task. The best results were found using accelerometers in multiple locations (both upper arms, both upper legs, sternum, and wrist of more affected side) and neural networks to analyze the data (40). Again, these multiple sensor locations are problematic for in-home monitoring of symptoms, though for short-term assessments they may be practical. Fortunately, similar results were also found using neural networks with a single sensor (tri-axial accelerometer and SEMG) on the wrist (31). The comparison suggests that machine learning algorithms capable of exploiting more features from fewer signals may be a critical enabler for balancing classification accuracy with user convenience. Such algorithms may be able to improve the generality of the promising gait-focused results found with a single waist-mounted tri-axial accelerometer (41). Perhaps there are features that could identify dyskinesia while the patient is at rest, or perhaps a middle ground of waist and wrist sensors could combine for that purpose. Wrist and waist sensors are thought to be the most acceptable sensor locations for the elderly.

Wearable sensors could be very useful in the detection of PD related presentations such as motor fluctuations and “on”/“off” states. To accomplish detection related goals in heterogeneous populations such as those with PD, most detection algorithms need to use an individual-specific detection threshold for each patient. This type of detection-related analysis is contingent upon training and calibrating the system to each individual and each sensor, which is a major practical challenge to any clinical deployment of this approach. The use of accelerometers on the sternum, wrist, and leg to compare bradykinesia, hypokinesia, and tremor symptoms did not lead to effective discrimination of “on” and “off” states (37). Promising results were found during walking using a waist-mounted tri-axial accelerometer (“off” vs. “on” with >90% sensitivity and specificity) (42, 44), but the gait-specific nature of the approach leads to limited applicability. To date, no sensor array has been able to detect and track on-off motor fluctuations in individuals with PD to a clinically meaningful extent. This failure may be due to the extreme heterogeneity of symptom presentation of individuals with PD, or perhaps the optimal configuration of sensors and algorithms has not yet been found.

Detecting FOG has proven difficult using only accelerometer frequency content because FOG episodes look very similar to standing still. Additionally, FOG is a symptom of PD that is not readily reproducible in the laboratory setting and often not fully understood by the individual experiencing the symptom. The standard method has been to place accelerometers on the ankles and to detect FOG based on a freeze index threshold (45, 46) plus a power threshold based on signal power in freeze and locomotor bands (47). However, the best results were found using individual thresholds, and individualized calibration is not ideal for wide use of a device as the measurement and implementation of participant specific algorithms is time intensive and not readily implementable in the clinical setting. Using a waist-worn accelerometer and a support vector machine algorithm, better detection accuracy was found compared to the frequency threshold methods, however this result was also found using patient-specific algorithms (52). Using more sensors such as a hybrid accelerometer and SEMG on the shin (48), or multiple accelerometer sensor locations (legs, wrists, chest, and waist) with machine learning algorithms (19) did not greatly improve upon results using sensors only on the ankles. The wrist location was especially investigated as an alternative to the ankle location, due to greater acceptance of a wrist sensor by the elderly, and using machine learning techniques they found an acceptable hit rate and specificity, but again by using individual parameters (50). Another recent development has been the use of temporal gait parameters to detect FOG (49). One study using a prototype device with IMUs on the ankles and back of the neck to track the transitions in the gait cycle showed that stride length decreased and cadence increased at the onset of FOG, suggesting feasibility of their method (49). Using this same principle, a complementary index freezing of gait criterion (FOGC) was created, based on continuous measurements of stride length and cadence from an IMU on the shank (51). This method performed better than the standard FI frequency method in detecting FOG events. Another recent paper used clinical observations of heel trembling and no forward movement to detect FOG based on trembling events detected using heel mounted IMU signals combined with information about the speed of the foot (53). They also observed better performance than the standard FI method both in accuracy detecting FOG events and lower false positive rates (53). Perhaps even better results could be found by combining frequency and gait parameters in machine learning algorithms.

### Moving In-home Monitoring Forward

The ability of wearable sensors to detect symptoms for both patient assessment and real-time intervention is a promising result to build on. However, this review also highlighted some limitations of previous studies on wearable sensor systems in PD. The number of sensors used for the best results (such as sensors on the arms, legs, sternum, and wrist to detect dyskinesias (40) would not be acceptable for long-term use in a home setting. In addition, for symptoms such as dyskinesia, “on”/“off” fluctuations, and freezing-of-gait, clinically significant results have only been shown using individual-specific detection thresholds, which are a serious barrier to practical deployment. The need for substantial training sets is a known limitation for machine learning algorithms, which is only exacerbated in this context by the need for the trainers to be expert clinicians. This cost might be acceptable if a concerted one-time effort could develop a comprehensive training data set, but such effort would be futile if the algorithms still require individual-specific parameters that require training for each specific patient during specific ADL/IADL task performance. Finally, this literature review shows that more work is needed to validate the systems during unscripted and unconstrained activities of daily living in the home rather than in laboratory settings. Only 12 of the 27 studies reviewed were done in a home or apartment-like setting, despite that being the ideal setting to observe ADLs and FOG. It is more challenging to perform home studies because they require video annotation or observation by a clinician to establish a standard assessment, but this is the only setting in which the systems can be truly tested. Future implementation of this type of sensor would require substantial input and buy-in from clinicians who could perform in-home ADL/IADL testing such as occupational therapists.

Unscripted movements performed in an uncontrolled environment by individuals whose symptoms often vary from hour-to-hour and day-to-day presents a challenge for verifying test-retest reliability and validity of different assessment tools. Both behavioral and symptom-related variations in task performance can affect the data, potentially obscuring objectively favorable psychometric properties. However, in-home testing has much higher face validity than highly structured and standardized laboratory testing, so the goal of demonstrating reliability is a worthy one. To establish concurrent validity, several of the reviewed papers compared the measured movement characteristics with observations from trained clinical raters based on either video annotation (19, 22, 29–32, 34–36, 38–43, 45–48, 50–52) or clinical scales like UPDRS (29, 30, 32, 33, 35, 36) or m-AIMS (30, 38–40, 43). Overall, these tests showed good correlation between objective measurements and clinical scores, especially when using machine learning algorithms. However, it was also brought up that poor correlation does not necessarily mean poor validity when comparing objective and subjective measurements because the sensors could be more sensitive to small changes in disease state than a clinician, and the movements performed during clinical exams are different from the movements performed during daily activities. As an alternative approach, several studies compared the measured movement characteristics with subjective self-assessment by the individuals (37, 44), but this approach is problematic because individuals are not always aware of their symptoms and do not record their state often enough for quantitative reporting. In addition, FOG presents a special challenge because FOG scoring is not standardized and experts show only moderate reliability (both individually and as a group) in identifying the number of FOG events in a given observation period (45). However, the percent of time “frozen” has shown to be a much more reliable measure; thus, the most reasonable standard at present may be based on this metric, perhaps using two or more observers who score the same videos (45). Future research should focus on identifying clear criteria for FOG onset and offset.

Test-retest reliability presents an even greater challenge. This is a crucial property if wearable assessments are to be used in ecologically valid environments. Yet, few of the studies reviewed provided any analysis of test-retest reliability. Most monitored motor symptoms during loosely-defined scripted activities and offered little or no repeat testing of individual participants. Many used a reasonable leave-one-out bootstrap validation of their machine-learning algorithms, and many reported performance using a conservative, non-subject-specific training set. However, these precautions do not guarantee the algorithms will perform as well in real life, especially since most were trained only during specific tasks. The only study to provide test-retest reliability data was Hoff et al. (38), which showed high reproducibility for the correlation of frequency characteristics in 1–4 Hz and 4–8 Hz bands with m-AIMS score while sitting in a chair and abstaining from voluntary movement (38). Though challenging, future studies should consider performing test-retest comparisons and other reliability characterization during both scripted and unscripted activities, to ensure confidence in their results and applicability in everyday scenarios. One of the ultimate goals of home-based monitoring must be to define the minimal set of sensors that can detect and describe the severity of all the common motor symptoms of PD. The sensors should be as unobtrusive as possible; approaches such as attaching them to watches and belts that patients already wear should be an ongoing focus. Since FOG occurs in the legs, best practices for attaching sensors to the legs should be explored, with feedback from patients. Approaches that require surface electromyography require special attention due to the need for reproducible electrode placement in a home setting by untrained personnel. These issues of patient burden are of paramount importance if long-term monitoring is to impact assessment and intervention in PD. Development of algorithms that accomplish significant degrees of self-training (such as DNNs) or that are successful without individualized parameters would also be a critical step forward.

Moving forward, IMUs appear to be the best technology to monitor individuals with PD. They can be manufactured at low cost and microcontrollers today are readily available that can handle the increased computational load compared to gyroscopes or accelerometers alone. The synchronized measure of linear acceleration and angular velocity to provide a stable estimate of sensor orientation as well as both linear and angular motion information seems to be especially advantageous in detecting complicated motor symptoms such as dyskinesias and FOG.

## Conclusions

Wearable movement and muscle activity sensors combined with machine learning algorithms have the ability to classify symptoms of PD during performance of ADLs and IADLs with clinically valuable levels of sensitivity and specificity. Different systems and algorithms can achieve the complementary goals of patient classification (for assessment) and real-time symptom detection (for intervention). However, the number and layout of sensors and the need to tune individual-specific detection thresholds currently limit the practical utility of these systems. Furthermore, different symptoms currently demand different sensors. Future research should focus on identifying a minimal set of wearables suitable for detecting a range of symptoms, on algorithms that are robust to individual variations, and on validation of the resulting systems in real home settings. IMU sensors and neural network algorithms appear to be the most promising tools moving forward.

## Author Contributions

HP, JT, and KP contributed conception and design of the study. JT performed the literature review and summarized the findings. JT, PA, and KP wrote sections of the manuscript. All authors contributed to manuscript revision, read and approved the submitted version.

### Conflict of Interest Statement

The authors declare that the research was conducted in the absence of any commercial or financial relationships that could be construed as a potential conflict of interest.
